# Boronic Acids of Pharmaceutical Importance Affect the Growth and Photosynthetic Apparatus of Cyanobacteria in a Dose-Dependent Manner

**DOI:** 10.3390/toxins12120793

**Published:** 2020-12-13

**Authors:** Emilia Niemczyk, Jerzy Pogrzeba, Agnieszka Adamczyk-Woźniak, Jacek Lipok

**Affiliations:** 1 Department of Pharmacy and Ecological Chemistry, Faculty of Chemistry, University of Opole, Oleska 48, 45-052 Opole, Poland; jacek.lipok@uni.opole.pl; 2 Department of Analytical Chemistry, Faculty of Chemistry, University of Opole, Oleska 48, 45-052 Opole, Poland; 118088@student.uni.opole.pl; 3 Laboratory of Synthesis and Structural Investigation of OrganoBoron Compounds, Department of Physical Chemistry, Faculty of Chemistry, Warsaw University of Technology, Noakowskiego 3, 00-664 Warsaw, Poland; agnieszka@ch.pw.edu.pl

**Keywords:** aryl boronic acids, pharmaceuticals, bactericidal activity, cyanobacteria, photosynthetic pigments

## Abstract

The dynamic increase in the commercial application of antimicrobial derivatives of boronic acids, and potential impact of their presence in aquatic systems, supports the necessity to study the toxicity of these substances towards microorganisms of crucial meaning in the environment. One example of the mentioned derivatives is tavaborole (5-fluoro-substituted benzoxaborole), a pharmaceutical agent with antifungal activity. Cyanobacteria were used as model organisms, which are photoautotrophic prokaryotes, as representative aquatic bacteria and photoautotrophs associated with the plant kingdom. To the best of our knowledge, we investigated this issue for the first time. In order to recognize the under-stress response of those microorganisms, the concentration of photopigments—a key factor in the activity of photosynthetic apparatus—was measured spectrophotometrically. We found that the 3-piperazine bis(benzoxaborole) significantly suppressed the growth of halophilic and freshwater cyanobacteria, at a concentration 3.0 mM and 0.3 mM, respectively. Our results also showed that the tested substances at micromolar concentrations stimulated the growth of cyanobacteria, particularly in the freshwater strain *Chroococcidiopsis thermalis*. The tested substances acted with various strengths, depending on their structure and concentration; nevertheless, they had a greater influence on the synthesis of phycobiliproteins (e.g., lowered their concentration) than on the formation of chlorophyll and carotenoids.

## 1. Introduction

**Figure 1:**
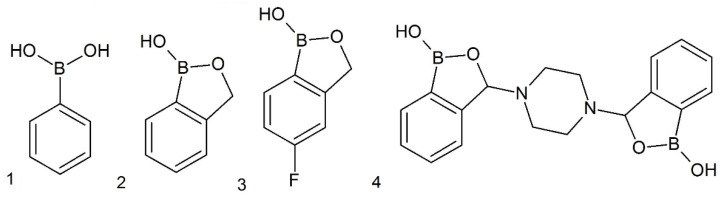
Structures of selected boronic compounds.

Boron does not occur as a pure element on Earth, but it is contained in detectable amounts in mineral connections (like oxides), for example, 5 mg per 1 kg in basalts, 100 mg per 1 kg in shellfish shells, and 4.5 mg dissolved in 1 L of ocean water. Boron, in physiological conditions, exists in freshwater primarily as nondissociated boric acid B(OH)_3_ (approximately 96%) and as borate anion B(OH)_4_^−^, its concertation in freshwater varies around 0.1–0.5 mg/L [1,2,3,4]. Nevertheless, this element was identified in the structure of the organic compound boromycin, which is a macrolide antibiotic produced by cyanobacteria belonging to the *Nostoc* genus; it is an effective agent against most Gram-positive bacteria [5]. Research on the role of organic derivatives of boronic acid in the metabolism of microorganisms showed that, depending on their structure, there can be different impacts on bacteria and fungi, ranging from stimulating their development to complete inhibition, or even death. Because of the high toxicity to microorganisms and lack of negative effects on mammals, boronic acid compounds are synthesized and tested for possible use as active constituents of antifungal and antibacterial drugs. The first results indicated that some compounds from this group, such as tavaborole (5-fluoro-substituted benzoxaborole) (Figure 1(3)), may be successfully used commercially in ointments that fight mycosis [6]. Its analogue 5-chlorobenzoxaborole with antifungal properties has recently completed the first phase of clinical trials, while benzoxaborole derivate possessing anti-parasitic activity is in the third phase of clinical trials [7,8,9,10]. The antimicrobial or antiparasitic mode of action of boron-containing compounds is mainly based on the inhibition of appropriative leucyl-tRNA synthetase, what leads to the blockade of protein synthesis. Moreover, boronic acids have also been identified as promising inhibitors of other enzymes—bacterial β-lactamase or methyltransferase and parasitic CPSF3 endonuclease, as well as inhibitors of efflux pump (NorA) [7,8,9,10,11,12,13]. Organic derivatives of boronic acid are a group of numerous and structurally disparate chemical compounds, which were expanded over the years with the synthesis of new compounds. One of the basic compounds of this group are phenylboronic acid (Figure 1(1)) and benzoxaborole (Figure 1(2)), which are the starting structures for the synthesis of more complex structures, such as 3-piperazine-bis(benzoxaborole), with different physicochemical parameters (Figure 1(4)). Due to the growing interest and extending spectrum of usage as potential therapeutics, aryl derivatives of boronic acids will, in the future, be present in aquatic environments. Many studies on the susceptibility of various species of fungi and bacteria have been performed, but whether derivatives of boronic acid influence cyanobacteria in any way has not yet been shown [9,10,14,15,16,17,18,19].

Cyanobacteria are Gram-negative photosynthetic bacteria that belong to the group of prokaryotic microalgae, which inhabit most known biocoenoses due to their wide species diversity and high adaptability to environmental conditions. They reside in fresh water, sea water, reservoirs of high salinity, and places with low humidity, and may even live on other organisms [20]. Cyanobacteria could be arranged in freshwater and halophilic species, according to their salt-tolerance. Halophiles are salt-loving organisms which live in hypersaline waters [21]. Among the many astonishing properties of these pioneering microbiota is the dynamic biosynthesis of many complex organic substances (e.g., sugars, lipids, and proteins) from simple inorganic salts, water, and carbon dioxide. Cyanobacteria also metabolize some organic substances that are toxic to other living organisms (e.g., organophosphorus compounds) [22]. To be effective primary producers and to simultaneously perform biotransformations of xenobiotics, cyanobacteria must obtain remarkable amounts of necessary energy. Blue-green algae collect this energy from sunlight via an extensive photosynthetic system built of thylakoids and phycobilisomes, which provides the ability to absorb visible radiation. Photosynthetic dyes, such as chlorophyll, carotenoids (in thylakoids), and phycobiliproteins (arranged in phycobilisomes), play key roles in this system [23,24]. The role of these pigments in co-creating the expended photosystems of cyanobacteria is crucial because this composed system allows these organisms to absorb the energy of light at ranges and amounts that are not available for plants and some microalgal species. When one or two pigments in the biosynthetic pathway are damaged, other pigments may take over the role of light energy acquisition [23,25,26]. Photosynthetic dyes of cyanobacteria protect cells from stress factors (reactive oxygen species and UV ratio) and may build a storage of chemical elements. The concentration of these pigments and the quantitative proportions between these substances are good markers that reflect the ability of autotrophic organisms to grow and maintain homeostatic stability in the environment, as well as in laboratory experiments [27].

Therefore, the physiological responses of cyanobacteria to the action of aryl boronic acids, with antifungal or antibacterial action, were studied in respect to photosynthetic dyes and microbial growth rate. This question is of special interest because cyanobacteria simultaneously representing bacteria and photoautotrophic organisms are the producers of boromycin—a natural antibiotic which contains boron [5].

## 2. Results

### 2.1. Growth of Cyanobacteria

Due to earlier proven significant differences in the sensitivity of halophilic and freshwater cyanobacteria to xenobiotics, the concentration ranges of aryl boronic acids varied by one order of magnitude. Lower values were used for freshwater strains, whereas higher values were used for halophilic strains [15,28]. The influence of two types of aryl boronic acid structures was reported: the first group had one benzoxaborole function (1, 2, 3) and the other group had two of these units (4). Each of the tested boronic compounds exhibited a different influence on the examined cyanobacterial strains.

**Figure 2:**
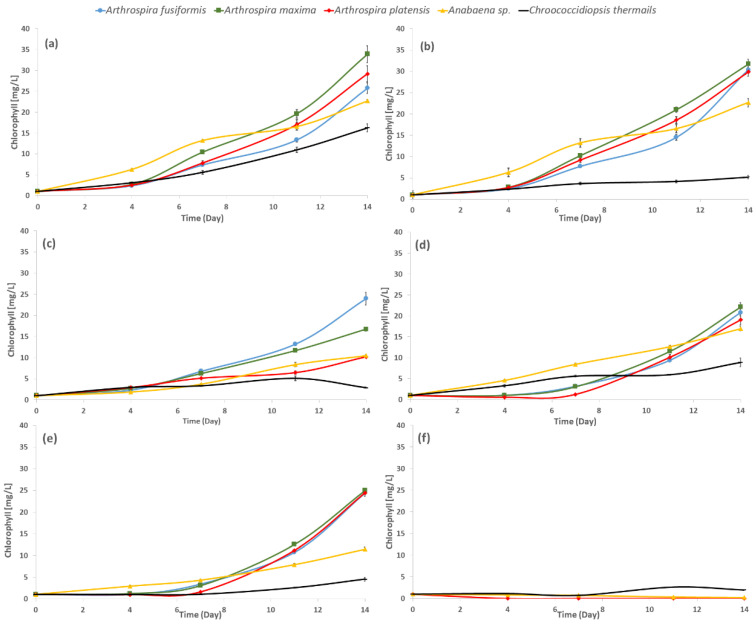
The influence of selected boronic compounds, at the concentrations of 3.0 mM for halophilic and 0.3 mM for freshwater species, on the growth of examined cyanobacteria in respect to appropriate controls: (**a**) Control cultivations without boronic acids and without DMSO; (**b**) control cultivations without boronic acids, but with DMSO; (**c**) phenylboronic acids; (**d**) benzoxaborole; (**e**) 5-fluoro-substituted benzoxaborole; and (**f**) 3-piperazine-bis(benzoxaborole).

Figure 2 presents the growth curves of tested photoautotrophs based on chlorophyll concentration, which is a determinant of metabolic activity of cyanobacteria [29]. The presented growth curves reflect the impact of tested compounds in the highest applied concentration (3.0 mM and 0.3 mM for halophilic and freshwater species, respectively). The growth rates were calculated from the slopes of the growth curves.

There were significant differences in the amount of chlorophyll among tested cyanobacteria. The impact of the solvent (DMSO) on the growth of tested cyanobacteria was noticeable, especially in the case of *Chroococcidiopsis thermalis*, where the presence of DMSO in the medium significantly inhibited the growth of this species. Hence, all experimental data (the influence of boronic acids) were compared to the control cultivation with DMSO. As a result, we confirmed that halophilic species generally exhibit a higher content of total chlorophyll, and the maximum amount was observed for *Arthrospira maxima*. The freshwater strain *Anabaena* sp. was characterized by a higher content of chlorophyll than *Chroococcidiopsis thermalis* (Figure 2a).

Although the influence of tested boronic acids on cyanobacterial growth varied depending on the structure, concentration, and strain, all tested halophilic *Arthrospira* species showed a similar response to the presence of phenylboronic acids (1), benzoxaborole (2)**,** and 5-fluoro-substituted benzoxaborole (3). The growth of halophilic strains was found to be suppressed until day 7, especially in the case of benzoxaborole and its fluoro derivative (Figure 2d,e). The growth of all halophilic species was completely inhibited after 4 days of culturing with 3-piperazine-bis(benzoxaborole) (Figure 2e). *Ch. thermalis* was found as the most resistant strain towards all tested boronic acids (Figure 2).

The most toxic compound was 3-piperazine-bis(benzoxaborole) (4), which inhibited the growth of all tested cyanobacteria at all applied concentrations in the most significant way, and totally stopped the growth of halophilic species at 3.0 mM and freshwater *Anabaena* sp. at 0.3 mM (Figure 2, Table 1). Only *Chroococcidiopsis thermalis* was an exception to the influence of this substance, as it stimulated its growth at 0.03 mM and 0.10 mM, and significantly, but not lethally, decreased growth at 0.3 mM (Figure 2, Table 1). The strong negative effect of (4) on cyanobacterial growth may be related to its structural features because two identical biologically active systems are inbuilt in one molecule. The influence of 3-piperazine-bis(benzoxaborole) was far more intensive compared to the impact of any compound that possessed one benzoxaborole function.

Notably, growth at the 1.0 mM dose of (4) was more limited for halophilic cyanobacteria than in the presence of any other tested compound at even the highest used concentration (3.0 mM). Phenylboronic acid (1) inhibited the growth of halophilic blue-green algae in a dose-dependent manner with increasing concentrations. A 10 times lower concentration of the (1) substance significantly stimulated the growth of freshwater cyanobacteria *Chroococcidiopsis thermalis*. The growth of another freshwater species *Anabaena* sp. was increased at 0.03 mM of substance (1). Benzoxaborole (2) showed a less harmful influence on halophilic microalgae than phenylboronic acid (1) in two cases, *Arthrospira fusiformis* at 1.0 mM and *Arthrospira maxima* at 0.3 mM, which stimulated the growth of the microorganisms. The (2) substance was structurally different from phenylboronic acid (1), which contained boron in the hetero ring and has one hydroxyl group. This difference may have caused the significant stimulation of growth of *A. fusiformis* and *A. platensis* at 1.0 mM and *Anabaena* sp. at 0.1 mM. The response of *Chroococcidiopsis thermalis* in the presence of benzoxaborole (2) in the medium was similar to phenylboronic acid (1). The presence of 5-fluoro-substituted benzoxaborole (3) in the environment of the cultivation showed interesting results. This substance showed a more toxic impact than benzoxaborole on *Arthrospira* fusiformis (at 1.00 and 3.00 mM) and *Anabaena* sp. (at 0.1 and 0.3 mM). The lower concentration (0.3 mM for halophilic and 0.03 mM for freshwater cyanobacteria) of (3) substance stimulated the growth of cells, except *Anabaena* sp.

The effect of substances (1), (2), and (3) showed similar trends on the tested species at concentrations below 3.0 mM (for halophilic) and 0.3 mM (for *Anabaena* sp.). Notably, one of the freshwater species, *Chroococcidiopsis thermalis,* showed an outstanding response to the presence of the tested boronic compounds. The growth rate of *Ch. thermalis* was several times higher (up to four times) for concentrations of the tested boronic acids below 0.3 mM compared to the control. Notably, *Ch. thermalis* showed higher growth rates in the presence of substances (1), (2), and (3) at concentrations of 0.3 mM compared to the halophilic species, but the stimulation effect was much less observed at the same doses of the tested substances (Table 1). This observation indicates that the higher tolerance of halophilic cyanobacteria species to chemical stressors was not a definite phenomenon [15,28].

### 2.2. The Concentration of Photosynthetic Pigments as a Factor of Change in Photosynthetic Apparatus

The results of the relationship between the particular photosynthetic pigments presented below as percentage values with respect to appropriative controls refer to the state on the last day of culture (14th day). The growth rate describes changes in growth development over 14 days of cultivation under stressor conditions.

Concentration of photosynthetic pigments in the cultivations without aryl boronic acids (controls) varied depending on the tested species. The total chlorophyll concentration in the 14th day of culturing was around 25 mg/L for *A. fusiformis*, 35 mg/L for *A. maxima*, 30 mg/L for *A. platensis*, 20 mg/L for *Anabaena* sp., and 5 mg/L for *Ch. thermalis*. The total PBP concentration was approximately 80 mg/L for *A. fusiformis*, 110 mg/L for *A. maxima*, 75 mg/L for *A. platensis*, 180 mg/L for *Anabaena* sp., and 80 mg/L for *Ch. thermalis.* The total carotenoids concentration was estimated at 6 mg/L for *A. fusiformis*, 8 mg/L for *A. maxima*, 9 mg/L for *A. platensis*, 5 mg/L for *Anabaena* sp., and 4 mg/L for *Ch. thermalis.*

**Figure 3:**
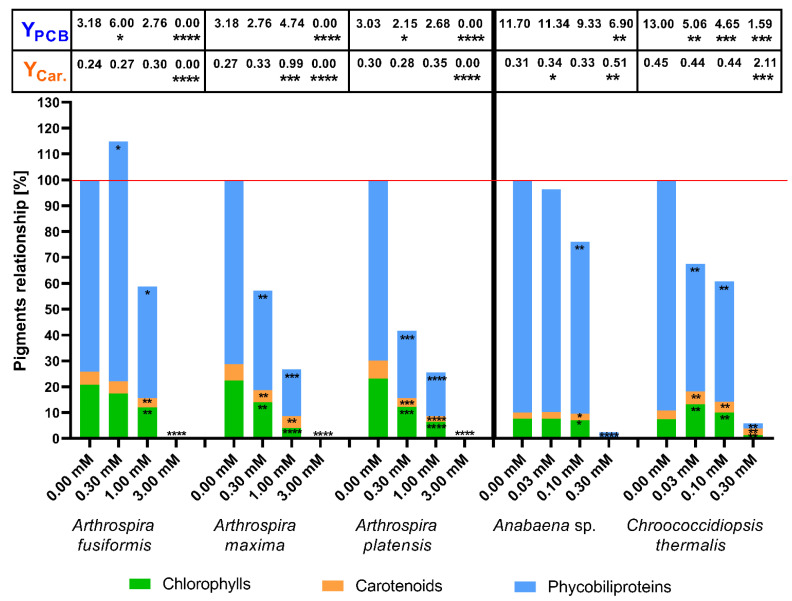
The effects of concentrations of 3-piperazine-bis(benzoxaborole) (4) on photosynthetic pigments in cyanobacterial cells. Pigment contents were measured on the 14th day after inoculation. The yield of phycobiliproteins (Y_PCB_) and carotenoids (Y_Car._) are presented in the table above the bars. The significance of the influence of 3-piperazine-bis(benzoxaborole) (4) on pigment content is shown in the form of asterisks (0.05 < *p*; * 0.0332 < *p* < 0.05; ** 0.0021 < *p* < 0.0332; *** 0.0002 < *p* < 0.0021; **** *p* < 0.0002) as tested against the appropriate controls. The maximum of standard error was around 9.8% and 9.0% for pigment concentration and yield, respectively.

The first presented results show the influence of (4) to pigment biosynthesis in the cells of the tested cyanobacteria on the last (14th) day of cultivation. The (4) substance was structurally different to the other three substances. This compound was constructed with two benzoxaborole units and one piperazine as a linker. The tested compound may be hydrolysed or undergo other complex reactions in the microbial cultures, which changed its properties. The results of the total amount of pigments are shown in Figure 3.

The results showed that the presence of 3-piperazine-bis(benzoxaborole) significantly decreased the amount of photosynthetic pigments in halophilic species of cyanobacteria because the tested substance concentrations increased. The exception was the influence of (4) at 0.3 mM on *A. fusiformis*, which increased the amount of phycobiliproteins observed. The halophilic strains completely stopped the production of pigments in the highest tested concentration (3.0 mM).

The correlation between the amount of pigments in cells and the concentration of the tested substance was not observed in the case of freshwater blue-green algae. An important decrease of pigments in *Anabaena* sp. was only noted at 0.1 mM of the (4) substance. Among the tested species, only *Chroococcidiopsis thermalis* demonstrated a positive response to the (4) compound. Doses of 0.03 mM and 0.10 mM of substance (4) significantly boosted production of chlorophylls and carotenoids in this species. Notably, *Ch. thermalis* cells also exhibited an inhibition of phycobiliprotein synthesis. The highest amount of the tested substance (0.3 mM) completely blocked the production of pigments in the halophilic species and freshwater *Anabaena* sp., and it only significantly stopped the biosynthesis of all determined pigments in *Ch. thermalis* (Figure 3).

**Figure 4:**
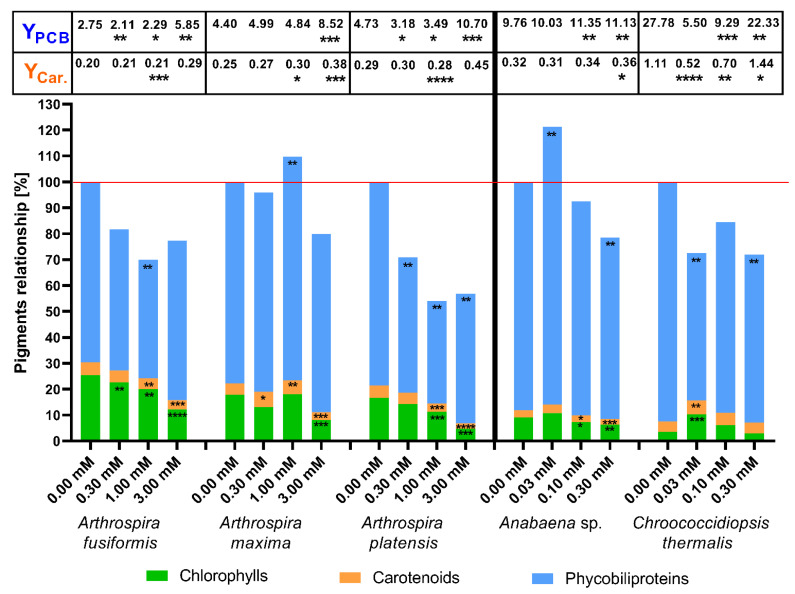
The effects of concentrations of phenylboronic acid (1) on photosynthetic pigments in cyanobacterial cells. Pigment content was measured on the 14th day after inoculation. The yield of phycobiliproteins (Y_PCB_) and carotenoids (Y_Car._) are presented in the table above the bars. The significance of the influence of phenylboronic acid (1) on pigment contents is shown in the form of asterisks (0.05 < *p*; * 0.0332 < *p* < 0.05; ** 0.0021 < *p* < 0.0332; *** 0.0002 < *p* < 0.0021; **** *p* < 0.0002), as tested against the appropriate controls. Maximum of standard error was around 9.6% and 9.2% for pigment concentration and yield, respectively.

A weaker influence was observed in the changes in the concentrations of photosynthetic pigments in the presence of phenylboronic acid (Figure 4), which built a smaller molecule (one phenyl ring substituted with one moiety of boron acid). The addition of phenylboronic acid (1) increased the content of chlorophylls on the 14th day at different concentrations in *A. maxima* (1.0 mM), *Anabaena* sp. (0.03 mM), and *Chroococcidiopsis thermalis* (0.03 mM and 0.1 mM) cells. The (1) substance significantly stimulated the production of carotenoids in three cases: at concentrations 0.3 and 1.0 mM in *A. maxima* and 0.03 mM in *Ch. thermalis*. The other cyanobacterial strains showed irrelevant changes in the quantity of total carotenoids in the presence of this compound. The highest used concentration of phenylboronic acid (1) significantly stimulated the specific biosynthesis of carotenoids in halophilic cyanobacteria and *Chroococcidiopsis thermalis*. Treatment of *Ch. thermalis* cells with 0.03 and 0.1 mM of this acid (1) caused decreased the carotenoid yield.

Two halophilic strains showed a strong reduction in phycobiliprotein production as a consequence of the 1.0 mM phenylboronic acid treatment. Strong inhibition of PBP biosynthesis was observed in *Arthrospira platensis* with the 0.3 and 3.0 mM doses of the tested substance. An important change in PBP production in *Arthrospira maxima* cells was observed at the 0.1 mM dose of phenylboronic acid. The presence of the 0.03 mM (1) compound in a cultivation medium induced an increase in the amount of PBP in *Anabaena* sp., but a significant decrease in *Chroococcidiopsis thermalis*. In the case of influence, the Phycobiliprotein production yield increased significantly at 3.0 mM phenylboronic acid in halophilic species. The concentrations of 0.03 mM and 0.1 mM of compound (1) intensively decreased synthesis efficiency in *Ch. thermalis* cells. The total amount of photosynthetic pigments in the presence of phenylboronic acid was higher than control samples (100%) only in halophilic *A. maxima* at 1.0 mM concentration and freshwater *Anabaena* sp. at 0.03 mM. These results showed that phenylboronic acid significantly reduced the concentrations of chlorophylls and carotenoids at the 1.0 mM dose in halophilic cyanobacteria species and at 0.1 mM in *Anabaena* sp. cells. Only in *Arthrospira maxima* cells (at 1.0 mM) and *Anabaena* sp. (at 0.03 mM), the (1) substance increased the total content of pigment that exceeded 100%. The main reason for these results was a significant increase in phycobiliprotein production. The presence of 0.03 mM phenylboronic acid stimulated growth and boosted chlorophyll and carotenoid biosynthesis in *Chroococcidiopsis thermalis*, but the synthesis of PBPs ceased (Figure 4).

**Figure 5:**
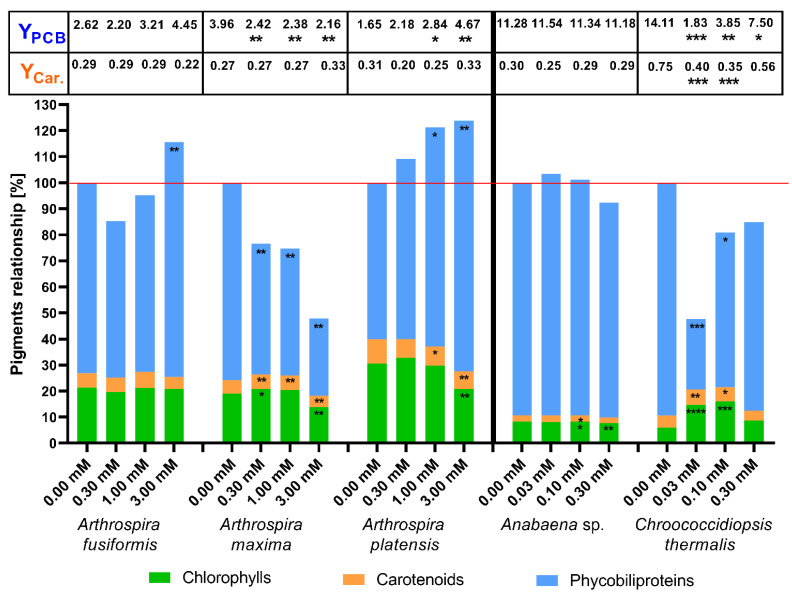
The effects of concentrations of benzoxaborole (2) on photosynthetic pigments in cyanobacterial cells. Pigment content was measured on the 14th day after inoculation. The yield of phycobiliproteins (YPCB) and carotenoids (YCar.) are presented in the table above the bars. The significance of the influence of benzoxaborole (2) on pigment content is shown in the form of asterisks (0.05 < *p*; * 0.0332 < *p* < 0.05; ** 0.0021 < *p* < 0.0332; *** 0.0002 < *p* < 0.0021; **** *p* < 0.0002), as tested against the appropriate controls. Maximum of standard error was around 9.7% and 9.8% for pigment concentration and yield, respectively.

The subsequent experimental substance was benzoxaborole, which differs from (1) in that it contains a boron atom in the heterocyclic ring (on one hydroxyl group). The substances (1) and (2) are the two basic units used to create a wide range of derivative boronic compounds. The difference between pigment relationships in the tested cyanobacteria cells and the influence of benzoxaborole are presented in Figure 5. Benzoxaborole had a different effect on the tested species of cyanobacteria than substance 3-piperazine-bis(benzoxaborole) and phenylboronic acid. Increased concentrations of substance (2) did not decrease the total amount of photosynthetic pigments (except *Arthrospira maxima*). On the 14th day of culture with benzoxaborole (2), significant stimulation of chlorophyll biosynthesis in cyanobacteria *A. maxima* (in case of 0.3 mM concentration) and *Chroococcidiopsis thermalis* (0.03 and 0.1 mM) was observed. A significant decrease in the amount of carotenoids in the presence of benzoxaborole was found in *A. maxima, Ch. thermalis,* and *A. platensis*. The first two species increased carotenoid synthesis at low and middle concentrations of the tested substances. The last species showed limited production of photosynthetic pigments in each experiment relative to control. Cultivation with benzoxaborole did not affect carotenoid production in any halophilic cyanobacteria or *Anabaena* sp. The 0.03 and 0.1 mM doses of the tested substance (2) inhibited the production of carotenoids in *Ch. thermalis* in a statistically significant way.

Increasing concentrations of benzoxaborole slightly intensified the production of phycobiliproteins by *A. platensis. Chroococcidiopsis thermalis* and *Arthrospira maxima* exhibited a significant decrease in phycobiliprotein concentrations in response to 0.03 mM and 1.0 mM treatments with this boronic acid (2). In the presence of benzoxaborole, the level of PBPs in *Anabaena* sp. cells was not changed at all tested concentrations. *A. fusiformis* and *A. platensis* cells synthesized more PBPs in the presence of 1.0 and 3.0 mM benzoxaborole in the medium. *A. maxima* and *Ch. thermalis* showed low biosynthesis efficiency of protein pigments at all concentrations of the tested substance.

The results showed a significant increase in the total amount of photosynthetic pigments in the presence of benzoxaborole in *A. maxima* (at 3.0 mM) and *A. platensis* (at 1.0 and 3.0 mM). An interesting effect of (2) activity was observed in freshwater species. *Anabaena sp.* was not sensitive to any of the used concentrations of benzoxaborole, but *Chroococcidiopsis thermalis* produced increased amounts of chlorophylls and carotenoids and decreased PBP concentrations at concentrations of 0.03 and 0.1 mM (Figure 5).

**Figure 6:**
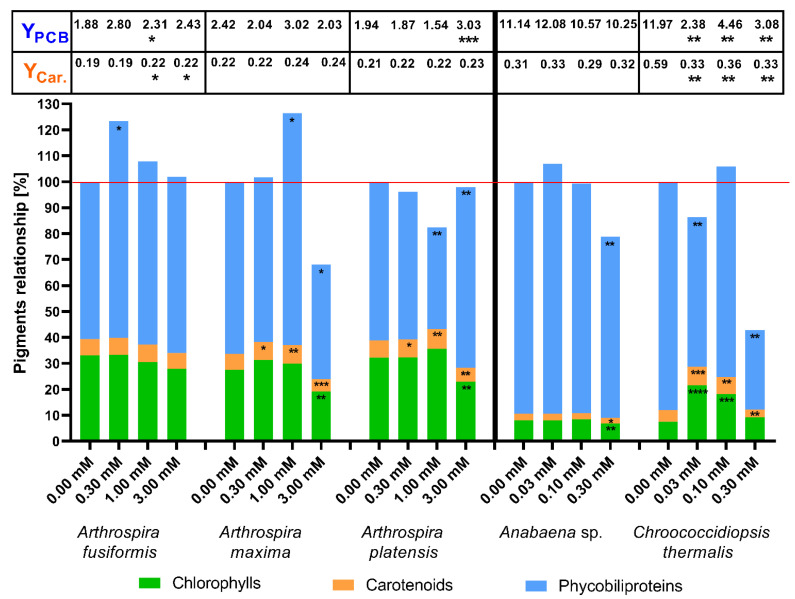
The effects of concentrations of 5-fluoro-substituted benzoxaborole (3) on photosynthetic pigments in cyanobacterial cells. Pigment content was measured on the 14th day after inoculation. The yield of phycobiliproteins (YPCB) and carotenoids (YCar.) are presented in the table above the bars. The significance of the influence of 5-fluoro-substituted benzoxaborole (3) on pigment content is shown in the form of asterisks (0.05 < *p*; * 0.0332 < *p* < 0.05; ** 0.0021 < *p* < 0.0332; *** 0.0002 < *p* < 0.0021; **** *p* < 0.0002), as tested against the appropriate controls. Maximum of standard error was around 9.9% and 9.8% for pigment concentration and yield, respectively.

The last tested substance was 5-fluoro-substituted benzoxaborole (tavaborole) (3), which possessed one fluorine atom substituted to the aromatic ring (Figure 6). (3) is used as an antifungal medicine. The experiments with 5-fluoro-substituted benzoxaborole showed similar effects as benzoxaborole (2). In the presence of increasing doses (0.3 mM and 1.0 mM) of (2) and (3), *A. maxima* and *A. platensis* significantly boosted their synthesis of carotenoids. Notably, treatment with 1.0 and 3.0 mM of (3) induced significant increases in specific yield of carotenoids in *A. fusiformis* cells. The biggest differences were observed in phycobilin production. The synthesis of these protein pigments was significantly stimulated in *A. fusiformis* (at 0.3 mM), *A. maxima* (at 1.0 mM), and *A. platensis* (at 3.0 mM). The 5-fluoro-substituted benzoxaborole at 0.3 mM stimulated the biosynthesis of PBP by *Arthrospira fusiformis*, but it inhibited this process in *Arthrospira maxima*. The 1.0 mM concentration of this compound caused opposite effects in *A. maxima* and *A. fusiformis* cells, which showed reduced and increased PBP production, respectively. Notably, compound (3) did not demonstrate a significant influence on phycobiliprotein yield in halophilic cyanobacteria, except at 3.0 mM in *A. platensis*.

The 5-fluoro-substituted benzoxaborole significantly decreased the content of pigments in *Anabaena* sp. at the highest tested concentration. The 0.03 mM and 0.1 mM doses significantly stimulated the production of chlorophylls and carotenoids in *Chroococcidiopsis thermalis* cells. The highest level of the examined substance (3) significantly inhibited the production of phycobiliproteins, and in the case of *Ch. thermalis,* it was on hold at the 0.03 mM dose. The total amount of pigment slightly exceeded the value of 100% in *Anabaena* sp. (at 0.03 mM) and *Chroococcidiopsis thermalis* (at 0.1 mM) (Figure 6).

We demonstrated that cyanobacteria are resistant to millimolar doses of boronic acids, and that these substances stimulate growth (Figure 2, Table 1) and the biosynthesis of photosynthetic pigments in some cases. Changes in PBP and carotenoid biosynthesis suggest that cyanobacteria possess various mechanisms to counteract the stress caused by the tested aryl boronic acids. Notably, the cyanobacteria *Chroococcidiopsis thermalis* demonstrated the highest carotenoid content, but the PBP rates and yield during treatment with boronic compounds were low. Our results showed that some millimolar concentrations of boronic compounds changed the metabolism of halophilic cyanobacteria towards higher phycobiliprotein production, but it did not always correlate with a higher PBP yield (Figure 3, Figure 4, Figure 5 and Figure 6). For the yield of phycobiliprotein synthesis, we demonstrated that the efficiency of carotenoid biosynthesis varied and depended on the concentration of the tested substances and cyanobacteria species.

## 3. Discussion

The influence of antifungals and antibacterial boron-containing organic compounds on cyanobacteria was not previously investigated. Our results showed that blue-green algae were resistant to the tested compounds in most cases and exhibited an increased growth rate during the development of the population in cultivation (Figure 2, Table 1). The structural features of the tested compounds, phenylboronic acids (1), benzoxaborole (2), and 5-fluoro-substituted benzoxaborole (3) were comparable to each other, but 3-piperazine-bis(benzoxaborole) (4) was structurally unique in the applied substances because it contained two benzoxaborole functions connected with a piperazine ring. Notably, 3-piperazine-bis(benzoxaborole) was the most harmful of the tested boronic compounds. The growth of the examined cyanobacterial species (except for *Chroococcidiopsis thermalis*) was significantly suppressed up to 1.0 mM and 0.1 mM for halophilic and *Anabaena* sp., respectively. The highest concentration inhibited the growth the most for the mentioned cyanobacteria. This result is similar to the tested fungi species presented by Wieczorek et al. [14]. These authors showed that the examined strains of fungi, *Aspergillus niger, Aspergillus terreus, Fusarium dimerum, Fusarium solani,* and *Penicillium ochrochloron* demonstrated sensitivity to the (4) substance. Structure–activity relationship studies of 5-fluoro-substituted benzoxaborole have shown that the five-membered ring of oxaborole is key in antifungal activity. Therefore, the fact that (4) showed the highest anticyanobacterial effect of the tested substances suggests that the occurrence of two benzoxaborole units is also responsible for this activity. Piperazine groups might be indicated in the structure of (4); however, its environmental impact was assessed as low, with EC50 being around 10 mM [30]. Additionally, preliminary investigation of piperazine activity showed no influence of its presence on the growth of cyanobacteria at concentrations as high as 7–10 mM for freshwater and 12–14 mM for halophilic strains, respectively [31]. An interesting exception was the response of *Chroococcidiopsis thermalis*, in which lower doses of compound (4) (0.03 mM and 0.10 mM) significantly stimulated cell growth. Among the microorganisms examined by Adamczyk–Woźniak et al. [32], only *Escherichia coli* was resistant to 3-piperazine-bis(benzoxaborole). The results obtained from experimental cultivations with the highest tested concentration of phenylboronic acids showed that halophilic species were more resistant than *Staphylococcus aureus* and *Candida tenuis* (growth inhibition at 0.5 mM concentration). Benzoxaborole and 5-fluoro-substituted benzoxaborole (tavaborole), used today as an active ingredient in antifungal pharmaceuticals, were harmful to the tested blue-green algae at 0.3 or 3.0 mM doses in freshwater and halophilic strains, respectively. This result suggests that fluorine, as the substituent, is toxic to prokaryotic microalgae, which was also suggested by Bhatnagar and Bhatnagar [33]. The (3) compound was less harmful to cyanobacteria than NaF because the dose of 3.0 mM of 5-fluoro-substituted benzoxaborole only stopped the growth of cells, and the fluorine ion completely destroyed the colonies of *Synechococcus leopoliensis* and *Oscillatoria limnetica* [33]. All five examined strains of cyanobacteria were significantly more resistant to the presence of (2) and (3) substances than *Escherichia coli, Staphylococcus aureus, Mycobacterium luteum*, *Candida tenuis,* and *Aspergillus niger*, because the 0.4 mM concentration of these substances (2) and (3) inhibited the growth of the tested bacterial and fungal species [14,16]. The exception discovered in our study was *Chroococcidiopsis thermalis*, which exhibited an increased growth rate in the presence of (2) and (3), even in the highest tested concentrations.

Cyanobacteria are resistant to many stress factors (like UV radiation, salt stress), including the influence of xenobiotics [34,35]. Some researchers [15,28] showed that blue-green algae were impervious to the presence of aminophosphonate in the environment and included these compounds in their metabolism. Their results showed that halophilic cyanobacteria were more resistant to aminophosphonate than freshwater species. The growth of the halophilic microalgae was stimulated by millimolar concentration of aminophosphonates. Freshwater cyanobacteria showed the same effect at a 10-times lower concentration of phosphonates, but the highest concentration was lethal [15,28]. However, our results showed the opposite dependence for the freshwater *Chroococcidiopsis thermalis*. In this case, a more intensive growth rate was observed in the presence of 0.3 mM of phenylboronic acid, benzoxaborole, and 5-fluoro-substituted benzoxaborole than the other halophilic strain in the same conditions. This effect shows that some freshwater cyanobacteria exhibit similar tolerance to chemical stress as halophiles. It is a very interesting observation because dependent on the types, cyanobacteria have a different mechanism to their response to stress factors. In general, halophilic cyanobacteria are more resistant due to very strict control of transportation processes between the inside and outside of the cell. Halophilic species showed an interesting response to phenylboronic acids, benzoxaborole, and 5-fluoro-substituted benzoxaborole in the highest tested concentration (3.0 mM). The growth of *Arthrospira* strains was slower in the first week of cultivation, and intensified in the rest of the time of the experiment compared to the control. These results suggest that the growth of halophilic cyanobacteria can be divided into two periods—adaptation to the new environmental conditions, followed by more intensive growth (Figure 2).

To indicate the changes in the photosynthetic apparatus under the influence of toxic microorganisms’ boronic acids, the compiled data of the concentrations of the main photosynthetic pigments on the 14th day of culture are presented. In most cases, the examined boronic acids decreased the synthesis of phycobiliproteins and carotenoids in *A. fusiformis* and *A. platensis* cells, but these processes were stimulated in some cultivations of *A. maxima*. The presence of boronic compounds in the medium significantly increased carotenoid concentration in *Chroococcidiopsis thermalis* cells, but caused degradation of phycobiliproteins in this species (Figure 3, Figure 4, Figure 5 and Figure 6). The higher amounts of chlorophylls in *Ch. thermalis* cells at the 0.03 and 0.10 mM doses of any tested substances confirmed earlier results of studies on plants, in which boron and its derivatives stimulated the growth and production of chlorophylls in plant cells [36]. The stabilization of cell wall proteins of plants by boron, which affects the charging of necessary substances from the environment, is well-documented. Boron creates a complex with proteins and carbohydrate (mainly associated with hydroxyl groups) and cross-links components of the cell wall. The presence of boron also supports the adsorption of atmospheric nitrogen in heterocystous species of cyanobacteria. The mechanism of action and role of boron in cyanobacteria is not well-known, but is dependent on the strain of these microorganisms [37,38]. Therefore, the higher growth rate of *Ch. thermalis* may be a result of a similar role of boron, as described for the plant cells described above. The available results show that some substances (like aminophosphonate) change the quantitative relationships between the pigments that compose the photosynthetic apparatus of blue-green algae, by reducing or increasing the amounts of phycobiliproteins and carotenoids in cells [15,28,34]. Notably, the total content of pigments was only higher than the control of *Ch. thermalis* for the (3) substance at a concentration 0.1 mM. The stimulating effect on pigment biosynthesis of (3) at this concentration applied to all of the measured groups of pigments. The total amount of pigments on the 14th day of cultivation of *Chroococcidiopsis thermalis* was lower than the control. A higher total concentration of photosynthetic pigments (than the respective control experiments) was also observed in some experiments with halophilic cyanobacteria. According to our results, a more intensive production of phycobiliproteins in halophilic cyanobacteria is responsible for this effect. Notably, the increased production of PBPs in the mentioned cases was accompanied by decreasing concentrations of chlorophylls and carotenoids. These results clearly show that cyanobacteria (depending on the species and tested compound) change the proportion between protein and non-protein photosynthetic pigments under specific environmental conditions. This observation also shows that some aquatic species of prokaryotic microalgae adapt to stress factors by more intensive growth, but other species (like halophilic cyanobacteria) need time to change their metabolism to enhance the production of photosynthetic pigments [15,28]. In this paper, we also calculated specific efficiencies of photosynthetic pigment production (yields), expressed as their content (in mg/L) per unit of chlorophyll (in mg/L). This factor gives further information on how cyanobacteria changes their pigment’s production under specific conditions, by correlation of pigment concentration with chlorophylls concentration. This method allows to better illustrate the effect of the tested substances to the specific production of pigments in cyanobacterial organisms. For example, in the case of *Anabaena* sp. (phenylboronic acids in 0.03 dose), we observed a statistically significant increase of the PBP amount, but changes in yield were not notable. This could mean that the increase in phycobiliproteins was caused because of more intensive growth. On the other hand, the same substance at concentration 3.00 mM did not change the concentration of total PBP in *A. maxima* cells, but increased the yield of this pigment in a statistically significant way. The same effect was observed with respect to carotenoid content, such as 1.00 and 3.00 mM of 5-fluoro-substituted benzoxaborole in the case of *A. fusiformis*. The changes in yields showed that the difference in amount of photosynthetic pigments in cyanobacteria in the presence of boronic acids is not just the result of changes in cell growth, but also the influence on cyanobacterial metabolism.

## 4. Conclusions

The impact of four aryl boronic compounds on the growth and composition of photosynthetic pigments in cyanobacteria was investigated for the first time. Among the tested compounds, 3-piperazine bis(benzoxaborole) (4), possessing not one, but two benzoxaborole units connected at their 3-positions with a piperazine ring, was the most active. The influence of tested boronic acids on the production of phycobiliproteins (which, in most cases, decreased their concentration) was greater than on the synthesis of chlorophyll and carotenoids. Structural features and dose-dependent toxicity towards cyanobacteria have been noticed. Our studies open the discussion on the environmental fate of pharmaceutically important aryl boronic acids, especially as potentially harmful xenobiotics in aquatic ecosystems. Cyanobacteria are considered the most common photoautotrophic organisms in biospheres, which may act as very good model organisms in toxicity studies due to their belonging to prokaryotes. Simultaneously, that microbiota may represent photoautotrophic organisms in environmental research. We showed that boronic acids changed the quantitative relationships between the content of chlorophylls, which are the main photosynthetic pigments of tested photoautotrophs, and the concentration of phycobiliproteins and carotenoids, to compensate for the efficiency of the photosynthetic apparatus. This observation indicates that some species of cyanobacteria can survive in harmful conditions due to heightened growth and modification of the quantitative relationship between components of the photosynthetic apparatus. The increasing interest of boronic acid compounds as promising pharmaceuticals entails the problem of the presence of their metabolites in wastewaters, and consequently, in aquatic ecosystems. Tavaborole conjugates, and metabolites are mainly excreted in the urine. Accumulation of compounds with biological activity may have harmful impacts on biodiversity or environmental balance. Moreover, the presented biological effect of tested boronic acids can be used as a base on studies on relationship between biological activity and molecular structures of these compounds. The influence of tested boron-containing compounds may be important to the pharmaceutical industry also because of new methods of application. The discovery of this phenomenon is a good starting point for more detailed experiments on the biochemical-level interactions of boronic acids and cyanobacterial cells, which can provide a better understanding of observed effects.

## 5. Materials and Methods

### 5.1. Tested Substances and Other Chemicals

All reagents used in our experiments and for cyanobacterial medium preparations, except benzoxaborole and its derivatives, were purchased from Sigma-Aldrich (Poznań, Poland). Benzoxaborole (2), 5-fluoro-substituted benzoxaborole (3) and 3-piperazine-bis(benzoxaborole) (4) were synthesized at the Faculty of Chemistry of Warsaw University of Technology by the research team of Prof. Agnieszka Adamczyk–Woźniak. Studies under aryl boronic acids stability showed that these compounds are stable under conditions of experiments (aquatic medium) [14,39].

### 5.2. Experimental Cultivations

The organisms used in the experiments included three halophilic cyanobacteria, *Arthrospira fusiformis* (CCALA 023)*, Arthrospira maxima* (CCALA 027)*,* and *Arthrospira platensis*, and two freshwater strains, *Anabaena* sp. (CCALA 007) and *Chroococcidiopsis thermalis* (CCALA 049). All species were obtained from the Botany Institute of the Academy of Sciences of the Czech Republic in Trebon. Cyanobacterial cultures were grown in sterile conical flasks with a capacity of 250 mL or 100 mL for halophilic or freshwater species, respectively. Cultivations were performed using liquid culture medium MSp (ATCC 1679) for halophilic species and BG11 (ATCC 616) for freshwater species. The stock (inoculum) cultures were revitalized every three weeks with the addition of a 10 mL aliquot of cells to 50 or 30 mL of fresh medium for halophilic or freshwater cyanobacteria, respectively. Microorganisms were grown at 25 ± 1 °C under 1050 lux lighting using a 16:8 photoperiod.

Experimental cultures were prepared by the addition of the proper amount of the tested boron compound that was previously dissolved in dimethyl sulfoxide (DMSO), and the final concentrations of the boronic compounds in the culture media were 0.30, 1.00, and 3.00 mM for halophilic species and 0.03, 0.10, and 0.30 mM for freshwater species. The literature supported a 10-times lower concentration of boronic compounds for freshwater cyanobacteria, which indicated that freshwater cyanobacteria were less resistant to stress factors than halophilic species [15,28]. The DMSO concentration in each sample was 50 µL in 30 mL of medium. Experimental setups were prepared in triplicate. Experiments were prepared as three independent restarts in three-week intervals. To determine whether DMSO affected the growth of cyanobacteria, two sets of control tests were prepared for each compound: (1) without the addition of DMSO and without the boron compounds, and (2) with the addition of DMSO but without the tested compounds. The experimental cultures were inoculated with the proper volumes of the stock cultures, and the initial concentration of chlorophyll was 1 mg/L in each culture.

### 5.3. Measurements of the Photosynthetic Pigments Chlorophyll, Phycobiliproteins and Carotenoids

The concentrations of chlorophyll, carotenoids, and phycobiliproteins were measured spectrophotometrically on days 0, 4, 7, 10, and 14 of cultivation in 1.4 mL special optical glass cuvettes using a Rayleigh 2601 UV-VIS spectrophotometer (Beijing, China). Chlorophyll and carotenoids extracts were prepared as follows. Cell aliquots (1 mL) were taken from the homogenized culture and precipitated via centrifugation at 13,000 rpm for 5 min, then 0.9 mL of the supernatant was discarded and the appropriate extraction solvent was added. Methanol was used for chlorophylls, and dimethylformamide (DMF) was used for carotenoids. The samples were shaken for 30 s and left for 10 min in the dark. This process was repeated twice. After extraction, the samples were centrifuged for 5 min at 13,000 rpm, and the clear solution above the precipitated cells was taken for UV-VIS measurements. Chlorophyll concentration was determined at 645 nm and 663 nm, and carotenoid content was established based on the absorbance measured at 461 nm and 664 nm. Dye concentrations were calculated using the following Equations (1) [40] and (2) [41]:

(1) $$C_{\mathrm{Chlorophyll}}[\frac{\mathrm{mg}}{l}]\;=\;20.2\;\cdot {\;A}_{645}+8.02\;\cdot {\;A}_{663}$$

(2) $$C_{\mathrm{carotenoids}}[\frac{\mathrm{mg}}{l}]\;=\;\left(A_{461}-\left(0.046\;\cdot {\;A}_{664}\right)\right)\cdot 4$$

To prepare samples for the determination of phycobiliproteins, 1.5 mL of the cell suspension was taken from experimental cultures and centrifuged for 15 min at 13,000 rpm in 4 °C. All supernatants were discarded, and 0.15 mL of glycerol was added to the remaining cells, mixed, and left for at least 24 h at −5 °C. Distilled water (1.35 mL) was added to the cells and mixed well. The obtained samples were centrifuged for 5 min at 13,000 rpm at 5 °C, and the clear supernatant solution was measured at 562 nm, 615 nm, and 652 nm. The concentrations of the appropriate phycobiliprotein dyes were determined using the following Equations (3)–(5) [42]:

Phycocyanin (PC)

(3) $$C_{\mathrm{PC}}\;\left[\frac{\mathrm{mg}}{l}\right]=\;\frac{A_{615}-(0.472\;\cdot \;A_{652})}{5.34}\cdot 1000\;$$

Allophycocyanin (APC)

(4) $$C_{\mathrm{APC}}\;\left[\frac{\mathrm{mg}}{l}\right]=\;\frac{A_{652}-\left(0.208\;\cdot {\;A}_{615}\right)}{5.09}\cdot 1000$$

Phycoerythrin (PE)

(5) $$C_{\mathrm{PE}}\;\left[\frac{\mathrm{mg}}{l}\right]=\;\frac{A_{562}-2.41\cdot \left[C_{\mathrm{PC}}\right]-0.849\;\cdot {[C}_{\mathrm{APC}}]}{9.62}\cdot 1000$$

Total concentration of phycobiliproteins (PBP) Equation (6):

(6) $$C_{\mathrm{PBP}}\left[\frac{\mathrm{mg}}{l}\right]{=C}_{\mathrm{PC}}{+C}_{\mathrm{APC}}{+C}_{\mathrm{PE}}$$

The specific efficiencies (yields) of photosynthetic pigment production were calculated as a ratio between the amount of cyanobacterial pigment (carotenoids or phycobiliproteins) and the concentration of chlorophyll (used as indicator of biomass of the cells), according to the Equation (7) given below:

(7) $$Y=\;\frac{\mathrm{total}\;\mathrm{carotenoids}\;\mathrm{or}\;\mathrm{phycobiliproteins}\;\mathrm{amount}\;[\frac{\mathrm{mg}}{l}]}{\mathrm{total}\;\mathrm{chlorophyll}\;\mathrm{concentration}\;[\frac{\mathrm{mg}}{l}]}$$

### 5.4. Determination of the Growth Rate Ratio

The growth of the examined cyanobacteria was based on time-course measurements of total chlorophyll content (at 3–4 day intervals) in experimental and control cultures. The growth rates were calculated from the slopes of growth curves, which were received by laying out the average chlorophyll concentration value basing on each replicated culture. Lastly, the value of the ratio of growth rates of experimental cultivations with regard to the growth rate of appropriative controls was determined. This operation allows to demonstrate differences in growth dynamics of tested cyanobacteria. The percentage values of the ratios of growth rates were presented in the results (Table 1).

### 5.5. Statistical Analysis

The calculated concentrations of photosynthetic pigments were used for statistical analyses. Each experimental setup (species cultivation with tested concentration of aryl boronic acids) was prepared in triplicate. A specific aliquot of sample was taken from each flask for the measurement of protein or non-protein pigments concentration. The results obtained from the measurements of the relevant arrangements were defined as the content of the pigments. The chlorophyll concertation was recalculated to the percentage value using linear regression, and the percent of standard error (SE) values was reported. To present changes in the content and proportion of specific photosynthetic pigments, their concentrations on the 14th day of cultivation were calculated as the percentage of the total content of pigment. The total content of pigment measured in the appropriate control experiments was considered 100%. Statistical significance was determined using the multiple t test and corrected via multiple comparisons using the Holm–Sidak method using GraphPad Prism 8.0.2. Significant differences (in tables and charts) are marked with asterisks (0.05 < *p*; * 0.0332 < *p* < 0.05; ** 0.0021 < *p* < 0.0332; *** 0.0002 < *p* < 0.0021; **** *p* < 0.0002).

## Figures and Tables

**Table 1:** Effect of increasing concentrations of boronic acids on cyanobacterial growth. The data are presented as ratios of the growth rates of experimental cultures with respect to the growth rate of appropriate controls (expressed as percentage values). Means ± standard errors are reported. Significant differences are marked with asterisks (0.05 < *p*; * 0.0332 < *p* < 0.05; ** 0.0021 < *p* < 0.0332; *** 0.0002 < *p* < 0.0021; **** *p* < 0.0002) versus the appropriate controls.

Substance	Concentration	*Arthrospira fusiformis*	*Arthrospira maxima*	*Arthrospira platensis*	Concentration	*Anabaena* sp.	*Chroococcidiopsis thermalis*
Phenylboronic acid (1)	0.00 mM	100.0 ± 1.7	100.0 ± 3.8	100.0 ± 3.9	0.00 mM	100.0 ± 3.2	100.0 ± 8.4
	0.30 mM	92.2 ± 2.4 **	75.8 ± 15.2*	89.1 ± 3.8 **	0.03 mM	122.7 ± 7.3 **	406.9 ± 2.1 ****
	1.00 mM	83.1 ± 3.8 ***	95.9 ± 6.7	64.5 ± 3.8 ****	0.10 mM	81.4 ± 6.2 **	224.9 ± 7.1 ****
	3.00 mM	53.0 ± 5.1 ****	42.4 ± 2.4 ****	25.3 ± 3.2 ****	0.30 mM	70.1 ± 3.5 ****	97.9 ± 9.8
Benzoxaborole (2)	0.00 mM	100.0 ± 3.6	100.0 ± 2.3	100.0 ± 5.6	0.00 mM	100.0 ± 0.9	100.0 ± 0.9
	0.30 mM	98.9 ± 4.5	105.8 ± 1.2 ****	103.8 ± 7.3	0.03 mM	98.8 ± 6.3	297.1 ± 3.0 ****
	1.00 mM	107.2 ± 4.4 **	101.5 ± 2.2	93.3 ± 2.4	0.10 mM	100.0 ± 2.2	330.3 ± 4.9 ****
	3.00 mM	91.5 ± 3.7 **	68.5 ± 3.9 ****	65.4 ± 6.4 ***	0.30 mM	93.5 ± 2.3 **	157.9 ± 8.9 ****
5-fluoro-substituted benzoxaborole (3)	0.00 mM	100.0 ± 2.9	100.0 ± 4.0	100.0 ± 1.8	0.00 mM	100.0 ± 6.9	100.0 ± 14.0
	0.30 mM	102.3 ± 2.3	114.7 ± 3.4 **	103.7 ± 4.7	0.03 mM	97.2 ± 0.6	338.9 ± 4.1 ****
	1.00 mM	92.8 ± 3.0	110.7 ± 7.9 *	110.6 ± 5.5 *	0.10 mM	102.5 ± 2.8	280.5 ± 4.0 ****
	3.00 mM	80.8 ± 2.8 ***	69.5 ± 1.7 ****	69.5 ± 0.9 ****	0.30 mM	84.5 ± 2.9 **	127.1 ± 7.5 **
3-piperazine-bis(benzoxaborole) (4)	0.00 mM	100.0 ± 4.1	100.0 ± 2.1	100.0 ± 4.4	0.00 mM	100.0 ± 1.3	100.0 ± 16.8
	0.30 mM	79.8 ± 5.5 **	57.6 ± 9.3 ***	47.7 ± 7.3 ****	0.03 mM	98.17 ± 3.6	200.9 ± 8.4 ****
	1.00 mM	51.5 ± 11.9 ***	16.1 ± 0.2 ****	20.3 ± 6.3 ****	0.10 mM	94.2 ± 3.1	142.3 ± 2.6 ***
	3.00 mM	0.0 ****	0.0 ****	0.0 ****	0.30 mM	0.0 ****	23.3 ± 4.2 ****
